# Toward a population-level public health approach to support families living under continuous traumatic stress

**DOI:** 10.3389/fpsyt.2026.1929102

**Published:** 2026-10-15

**Authors:** Abigail H. Gewirtz, Mor Keleynikov, Hadar Eliash-Fizik, Nehama Lewis, Jessica Gale, Shir Itzhaky-Wruble, Adena Hoffnung-Assouline, Anastasia Viner

**Affiliations:** 1 Hebrew University of Jerusalem, Paul Baerwald School of Social Work & Social Welfare, Jerusalem, Israel; 2 Arizona State University, Tempe, AZ, United States; 3 University of Haifa, Haifa, Israel

**Keywords:** children, continuous traumatic stress, parenting, population health, public health messaging, selective prevention, universal prevention

## Abstract

Since October 2023, families across Israel have been living under conditions of continuous traumatic stress (CTS): ongoing, unresolved threat due to repeated rounds of regional conflict. Demand for mental health support has far outstripped the capacity of clinician-delivered services, leaving many families without evidence-based help. This community case study describes the development and early implementation of ADAPT-Israel. To our knowledge, this is likely to be the first population-level, parenting-centered public health approach designed for families living under CTS. The initiative uses a tiered model to provide parenting support across layers of care: (i) a universal layer of brief, trauma-informed public health messages and a self-directed, interactive online parenting program (with an optional AI avatar guide); (ii) a selective layer of facilitator-delivered ADAPT groups for live higher-risk families, beginning with families of long-serving military reservists; and (iii) an indicated layer of individual telehealth support for families experiencing clinical levels of difficulty. The approach adapts Adaptive Parenting Tools/ADAPT, an evidence-based program grounded in Social Interaction Learning theory and validated in a different context – for military families in the United States across three randomized controlled trials with nearly 1,000 military families. We describe the rationale, the methodology used to develop each tier, and early implementation experience. Feasibility and acceptability indicators from initial pilot groups are promising, including high group completion and positive participant reports of improved parenting and family relationships, though these are small-sample, self-report, early-stage findings rather than effectiveness evidence. We discuss practical implications, lessons learned, and the model’s potential utility in settings of protracted conflict and chronic threat.

## Introduction

Continuous traumatic stress/CTS refers to the experiences of large populations exposed to continuous and ongoing wars, armed conflict, terrorism, and/or chronic community violence, through which individuals face repeated traumatic events and persistent uncertainty regarding future safety (1, 2). CTS is particularly concerning for children and adolescents, as children exposed to ongoing threats show both short and longer-term elevated rates of emotional and behavioral difficulties, including posttraumatic stress symptoms, anxiety, sleep disturbances, and impairments in emotion regulation and executive functioning (2–4).

Beyond its impact on individual well-being, prolonged exposure to CTS can disrupt family functioning and undermine the caregiving systems that support healthy child development (5–7). As CTS affects entire communities rather than isolated individuals, its consequences extend beyond clinical mental health concerns and represent a substantial public health challenge for children and families living in conflict-affected settings (1, 2).

When CTS occurs across large communities or populations, intervention needs become highly heterogeneous. Families differ substantially in their levels of exposure, existing resources, and psychological responses, suggesting that no single intervention is likely to meet the needs of all affected families (8, 9). Consequently, prevention scientists have argued that public-health approaches should provide interventions that vary in intensity according to need (10).

Across diverse contexts of war, disaster, and political violence, the caregiving or parenting environment has emerged as one of the most consistent predictors of children’s adjustment following adversity (5–7). Parents scaffold children to regulate their emotional responses to threat, maintain routines and a sense of safety, interpret stressful events, and access social and practical resources (11). Through these processes, supportive parenting can buffer the impact of trauma exposure on children’s emotional and behavioral functioning (5, 7, 12). Parenting is thus a particularly promising lever for population-level intervention under these conditions (13). Because parenting support is generally non-stigmatizing, can be delivered in varied formats and intensities, and benefits children and parents at the same time, we posit that it offers an accessible entry point for a tiered public health response, as we describe below.

Public health approaches to parenting are relatively recent, following nearly 100 years of effective public health campaigns focused on a variety of threats to morbidity and mortality including infectious disease (e.g. polio), personal safety (car safety belts), and risk behaviors (e.g. alcohol and substance use, safe sex) (e.g., 14, 15). Public health approaches to parenting have predominantly been established in European countries (UK, Ireland, Netherlands, Norway, Sweden) and Australia (13, 16). More recently, however, the Global Parenting Initiative (a UK-based nonprofit) has implemented population-based approaches to parenting support in a range of countries in the Global South (17).

A reference librarian-assisted comprehensive search of the peer-reviewed literature revealed no extant example of a public health approach to supporting families in the context of CTS. However, it is evident that when services are fragmented, as is the case when a coherent public health approach is not available, many families go without any supports, some families receive substandard care (that is not evidence-based) and other families receive the wrong ‘dose’ of support – more or less than they need or want (10, 13). This challenge (a mismatch or lack of resources) becomes particularly salient and high stakes when the population as a whole is under severe stress because of the well-documented detrimental impacts of CTS on children through parenting (5, 18).

This paper, then, describes an innovative approach to supporting families in contexts of CTS via the development and implementation of a public health approach to effective parenting. The approach we describe, known as Adaptive Parenting Skills for Israel/ADAPT-Israel, incorporates both low-dose and low-cost but high reach ‘light touch’ supports, along with more intensive programming for families with greater needs living under conditions of CTS.

## Context in which the innovation occurs: setting and population

Since October 7^th^, 2023, Israeli families have been living under conditions consistent with CTS (19). On October 7, 2023, Israel experienced the single most deadly attack on its citizens since the founding of the state in 1948; more than 1200 men, women and children were killed by Hamas terrorists, and more than 200 were taken hostage into Gaza (20). Considering the proportion of the population killed, injured or taken hostage on one day, the October 7 attacks were approximately 13 times more deadly than those of 9/11/2001 in the United States (21). The attack was followed by an extended period of ongoing threat characterized by wars between Israel, Iran and Iranian proxies including Hamas in Gaza, the Houthis in Yemen, and Hizbollah in Lebanon. The period from October 7, 2023, onwards has been characterized by repeated missile attacks, large-scale military mobilization, displacement of populations from border regions, death and property destruction and persistent uncertainty regarding personal and national safety (22). As a result, the Israeli population has experienced elevated levels of psychological distress, with studies documenting substantial increases in symptoms of anxiety, depression, and posttraumatic stress across the population (23, 24).

Children and adolescents are particularly vulnerable, with many exposed directly or indirectly to violence, loss, military deployment of family members, and chronic disruption to daily routines (12, 19, 25, 26). Such conditions place considerable strain on family functioning while simultaneously increasing children’s reliance on caregivers as a primary source of safety, stability, and emotional support. Indeed, research conducted in contexts of war and political violence consistently identify supportive parenting as important protective factors for child adjustment (7, 27). The wartime family stress model further specifies these processes, positing that war-related stressors affect children both directly, and indirectly, through their impact on parents’ mental health and their parenting, so that supporting parents becomes a primary route to protecting children (6).

Given its central role in children’s adaptation to adversity, and its malleability, parenting represents one of the most robust and modifiable targets for intervention (28). Supporting parenting may confer a dual benefit: buffering children against the negative effects of trauma exposure while simultaneously improving parents’ own wellbeing and mental health (18, 29, 30). Parenting support also offers an accessible point of entry for families who do not reach specialty mental health services, either because they do not require clinical treatment or because they do not seek it (31).

The scale of need created by CTS presents a challenge for traditional mental health systems. When large segments of the population are affected simultaneously, clinician-delivered services alone are unlikely to meet demand. Indeed, by mid-2024, six months after the outbreak of the war, approximately one in three Israeli adults, more than three million people, were estimated to be experiencing significant mental health difficulties, including over 600,000 individuals in severe psychological distress (32). Children have similarly been affected, with referrals to child mental health services increasing by nearly 20% during the first year of the war (33). At the same time, Israel’s mental health system has struggled to absorb this surge in demand, with public-sector waiting times lasting months to a year and only a small proportion of affected families receiving professional support (32). These realities highlight the need for intervention models capable of reaching large numbers of families efficiently, before difficulties escalate to the level of specialist mental health care.

Population-level approaches to family and parenting support implemented across large regions or countries conceptualize parenting support as a resource that benefits public health, promoting child well-being and preventing the emergence of later behavioral and emotional problems (34). One influential example is the Triple P (Positive Parenting Program), a multilevel system implemented nationally in Australia through the government’s Parenting Education and Support Program (13). Triple P provides universal (online), selective, and indicated parenting support through diverse delivery channels, including healthcare services, schools, workplaces, and media platforms (13). By matching intervention intensity to family needs and maximizing reach, population approaches such as Triple P illustrate how parenting support can function as a population-level strategy for promoting resilience and preventing mental health difficulties.

At the universal level, public health or population level approaches provide the lightest touch interventions, such as messaging campaigns. Public health messaging campaigns have a long and well-evidenced record as tools for shifting population-level knowledge, attitudes, and behavior, drawing on the discipline of social marketing, which applies principles such as audience targeting and formative research to positive health outcomes (35). Within the parenting field specifically, media-based messaging has demonstrated meaningful impact (36). Despite this evidence, we could find no parenting-focused messaging developed for parents raising children under CTS. This gap is what the universal level of the ADAPT-Israel initiative is designed to address.

Although parenting interventions have typically focused on helping parents manage their children’s behavior, trauma and chronic adversity can undermine the emotional regulatory capacities that support effective parenting (5, 18). Exposure to ongoing stress is associated with elevated emotional distress, impaired self-regulation, difficulties in emotion regulation, and disruptions in family functioning, all of which interfere with parents’ ability to provide consistent and emotionally responsive care (5, 6). Consequently, parenting supports designed for trauma-exposed families incorporate strategies that strengthen parental emotion regulation alongside traditional parenting skills including effective communication with children, teaching with encouragement, warmth, problem solving, monitoring and limit setting (18).

Theory-based parenting programs have a strong evidence-base, having been validated across diverse populations over the past 30–40 years (37). Across the broader parenting intervention literature, randomized controlled trials of diverse theory-based parenting programs such as the New Beginnings program for divorce (38), Triple P (39), Incredible Years (40), and GenerationPMTO (41) have shown benefits to parenting, parental and child mental health lasting as long as 25 years following the intervention (28, 37). Countries that have implemented population health approaches to parenting have typically incorporated multiple tiers of evidence-based parenting interventions. These programs may be offered in different formats and at different levels of intensity and optimally acknowledge both family needs (for less vs. more intensive programs) and parent preferences (for different kinds of program formats (10, 13). With rapid progress in technology, available formats, dosages, and content of parenting interventions have expanded significantly (42). For example, while online, self-directed parenting programs have been available for at least 15 years, the development of chatbots, AI agents, and other AI material (e.g., AI videos; live avatar support) have expanded the possibilities for both delivery and interactivity. Similarly, facilitator-delivered formats are now available via both face-to-face and telehealth options, as well as in group or individual virtual and face-to-face versions (43). The availability of parenting interventions in virtual formats has also vastly increased access to such programs; 20–30 years ago, families were limited by what parenting supports or resources were offered by local clinics, schools, libraries or community centers. In the context of continuous traumatic stress, maximizing access to programming is crucial as families may be dealing with multiple, ongoing stressors including balancing work, parenting, military service, and disruptions in children’s schooling, transportation and other infrastructure during periods of active war.

Below, we describe the first population-level approach to supporting parenting and family resilience under conditions of CTS. We describe key elements of the program, the methodology used to construct the approach, and early findings regarding feasibility and acceptability.

## Key elements of the initiative

ADAPT-Israel extends the original ADAPT intervention beyond a multi-format evidence-based parenting program into a coordinated public-health system designed to provide parenting support across varying levels of family need. The Israeli initiative incorporates (a) universal trauma-informed messaging, (b) a self-directed online parenting program, and (c) facilitator-delivered group and individual interventions. The initiative builds on ADAPT, an evidence-based trauma-informed parenting intervention originally developed for U.S. military families, while extending it into a population-level framework adapted contextually and culturally - for families living under continuous traumatic stress in Israel. ADAPT is grounded in Social Interaction Learning/SIL theory, originally known as Coercion Theory and developed by Gerald Patterson (44). SIL holds that coercive parenting practices are key mechanisms through which environmental stress negatively shapes children’s adjustment, and that positive parenting skills are therefore targets for intervention (45, 46). Building on this foundation, ADAPT teaches a set of core positive parenting practices, including skill encouragement, effective (non-corporal) limit setting, emotion socialization and coaching, family problem-solving, monitoring, and positive involvement, and infuses each with mindfulness and emotion-regulation content intended to help parents manage their own distress so that they can parent effectively under conditions of ongoing stress (18).

ADAPT was originally developed for U.S. military families experiencing deployment-related stress and has subsequently been tested in multiple formats, including multi-family groups, individual sessions, telehealth, and self-directed online delivery (29, 43). Across three randomized controlled trials involving nearly 900 military families, ADAPT consistently improved parenting practices, parental wellbeing, and child adjustment, while demonstrating feasibility and effectiveness across delivery formats (18, 30, 42, 43, 47–50). Findings from these trials further suggest that different delivery formats may be particularly beneficial for families with different levels of need, providing empirical support for a flexible, preference-matched approach to intervention (51, 52). This body of work establishes ADAPT as a rigorously tested, format-flexible program for war-affected families, and provides the evidence base and delivery infrastructure on which ADAPT-Israel builds.

ADAPT-Israel first and foremost modifies the U.S. intervention for families living in a very different context and diverse culture. Although a detailed description of the nature of the modifications is beyond the scope of this paper, they have followed the theoretical and practical approaches previously used in other modifications of ADAPT (53) including addressing language, cultural and religious norms and metaphors, and the CTS context (safety and danger of not only the service member but also the entire family). For example, the ADAPT-Israel online program, detailed further on, includes parenting video vignettes showing Arab and Jewish families, religious and secular families, Moslems and Christians and the multiple races and ethnicities of the population of Israel including those of Middle Eastern, Caucasian, and African descent.

Second, ADAPT-Israel extends the format-flexible ADAPT program into a tiered public-health framework tailored to families living under CTS by adapting both the program and its approach for the context of CTS, the diverse Israeli culture, and by adding a low-touch, potentially high-reach brief messaging universal layer. **Figure 1** depicts this public health approach. The initiative thus provides four different products at three levels of support that vary in intensity and delivery format according to family needs and preferences. At the universal level, we are developing (i) a set of trauma-informed public health parenting messages, and (ii) a self-directed online program. At the selective level, ADAPT-Israel provides (iii) facilitator-led group interventions, and at the indicated level (iv) individual telehealth support. All supports are linked by a common theoretical foundation and shared parenting principles. In doing so, ADAPT-Israel translates the evidence base established in the United States into a scalable population-health model designed for the realities of ongoing conflict.

**Figure 1:**
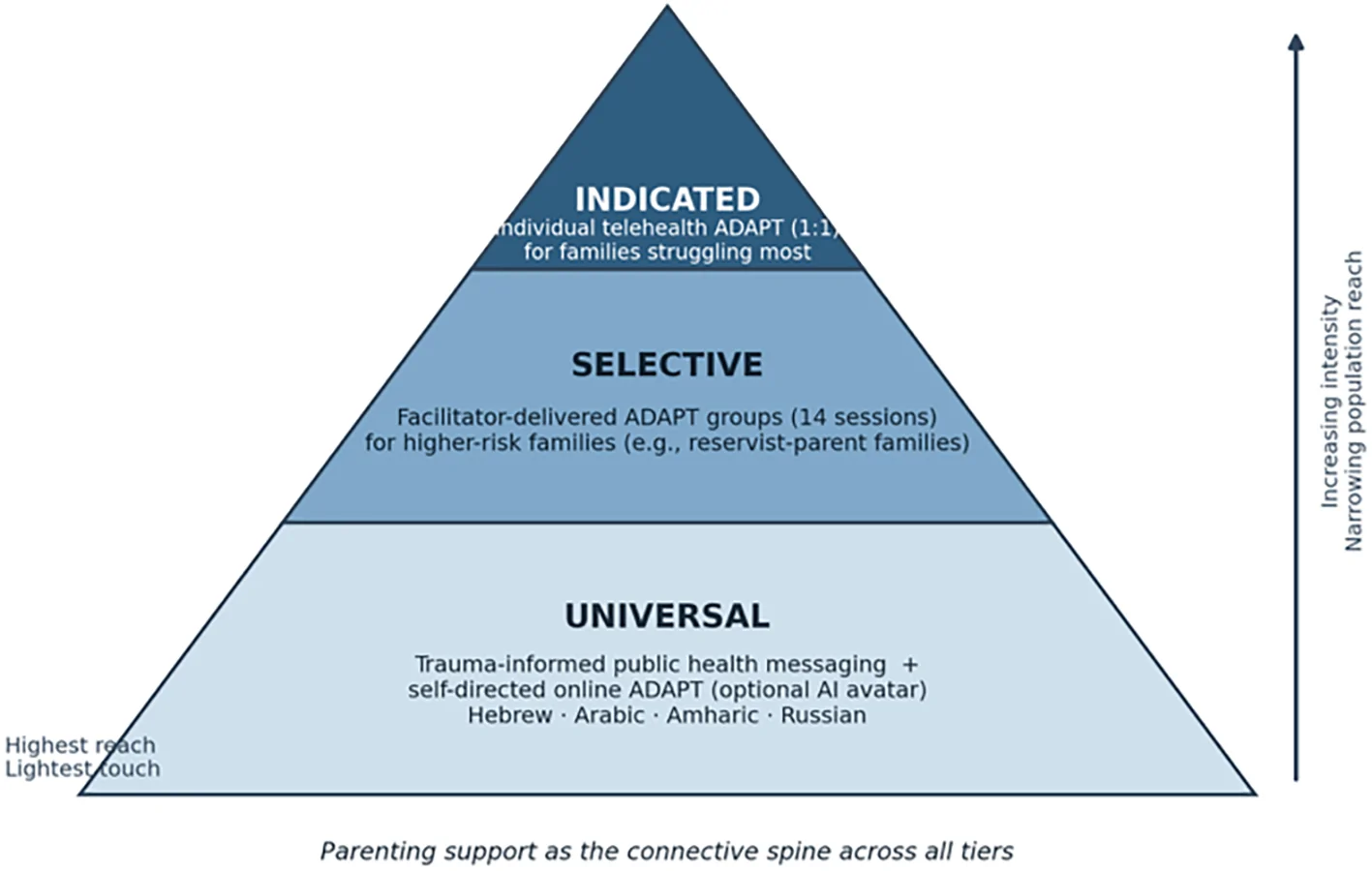
The ADAPT-Israel tiered public health model for families under CTS.

### Universal trauma-informed messaging: an audience-centered development process

At the universal level, the ADAPT-Israel initiative incorporates two components: public health messages, and a more intensive self-directed ADAPT online parenting program. The first layer involves the development of brief, accessible, trauma-informed public health messages for parents raising children under conditions of CTS. This component was designed as a low-intensity, high-reach layer of support. The goal is to provide parents with immediately usable guidance that normalizes distress, strengthens parental confidence, and connects families to additional supports when needed. Consistent with audience-centered and tailored health communication approaches, the message development process began with formative qualitative work intended to ensure that the content, tone, format, and dissemination channels were grounded in parents’ lived experiences rather than in generic, top-down parenting advice (54–56). At the time of writing, formative data collection has been completed, while thematic analysis and message development are underway. The messages have not yet been disseminated or evaluated.

The formative phase consisted of ten focus groups with diverse groups of Israeli parents living in different contexts of war-related stress and disruption. The groups were conducted between mid-2025 and mid-2026 and included 107 parents, with 6 to 16 participants in each group. Participants were drawn from diverse Jewish, Druze, and Muslim Arab communities, and the groups were conducted in participants’ primary language, Hebrew or Arabic. Focus groups were held with Druze families in a town along Israel’s northern border, in two Moslem Arab towns, and with Jewish Israelis in Jerusalem, Tel Aviv, Tiberias and in four smaller communities in the country’s north and center. The focus groups varied in population and setting but all followed a structured guide with ten open questions designed to elicit parents’ practical communication needs: what they find difficult to explain to children, which child reactions they struggle to interpret, what types of professional advice feel useful or alienating, which sources they trust, and where they would realistically encounter support during periods of high cognitive and emotional load. All focus groups were audio recorded and thus far seven focus groups have been transcribed. Six have been analyzed thematically, one is currently under analysis, and the remaining three are being transcribed. Following Braun and Clarke’s (57) thematic analysis approach, each of the six transcripts with analyses complete was analyzed independently using inductive coding by two research assistants. Following the analysis of each group, a group-level summary report was prepared, with responsibility for drafting the reports alternating between the two research assistants. The reports synthesized the themes and issues identified by both analysts. Given the applied goal of developing public health messages, coding focused not only on parents’ descriptions of distress, but also on the communication needs and potential message functions implied by their accounts, including normalization, reassurance, emotion coaching, practical scripting, help-seeking guidance, and connection to community and professional resources.

Preliminary findings from this ongoing formative analysis are being translated into a message map that links parent-identified needs to specific message themes, intended audiences, tone, delivery format, and dissemination channels. For example, themes related to parental guilt and uncertainty are being translated into messages that normalize common stress responses and emphasize that small, consistent parental actions can support children’s sense of safety. Themes related to difficult conversations with children are being translated into brief scripts that model what parents can say, how they can say it, and how responses may differ by child age. Themes related to symptoms of posttraumatic stress are being translated into guidance on what child stress reactions to watch out for in conditions of CTS, when additional support may be warranted, and how to seek help without framing help-seeking as a sign of parental (or child) failure. To date, the preliminary analysis has informed six draft message scripts addressing: tension between partners who respond differently to ongoing threat; community support as a coping resource; responding to children’s unexpected questions about death and loss; regression and emotional difficulties among older children and adolescents; hypervigilance and trauma-related triggers; and children’s renewed requests to sleep with their parents because of fear or nightmares. Four additional messages are planned and will be developed after the remaining focus group data has been analyzed. Across these message categories, the guiding principles are to be brief, concrete, non-stigmatizing, culturally responsive, and trauma-informed, emphasizing safety, trust, empowerment, choice, and connection.

The translation from themes to messages is being carried out iteratively in collaboration with trauma and parenting experts, community partners, and a digital communications team. The team entered into a collaboration with Compedia, an educational digital media company, to translate the draft scripts into animated videos. An animated format was selected in response to ethical and resource considerations. The first prototype addresses tension between partners when one remains highly anxious and vigilant while the other is attempting to return to routine. An initial version was reviewed by the project’s message design team, comprising a health communication researcher and two research assistants with backgrounds relevant to health and strategic communication, and is currently being revised by Compedia. **Figure 2** presents a static, illustrative adaptation of this draft vignette.

**Figure 2:**
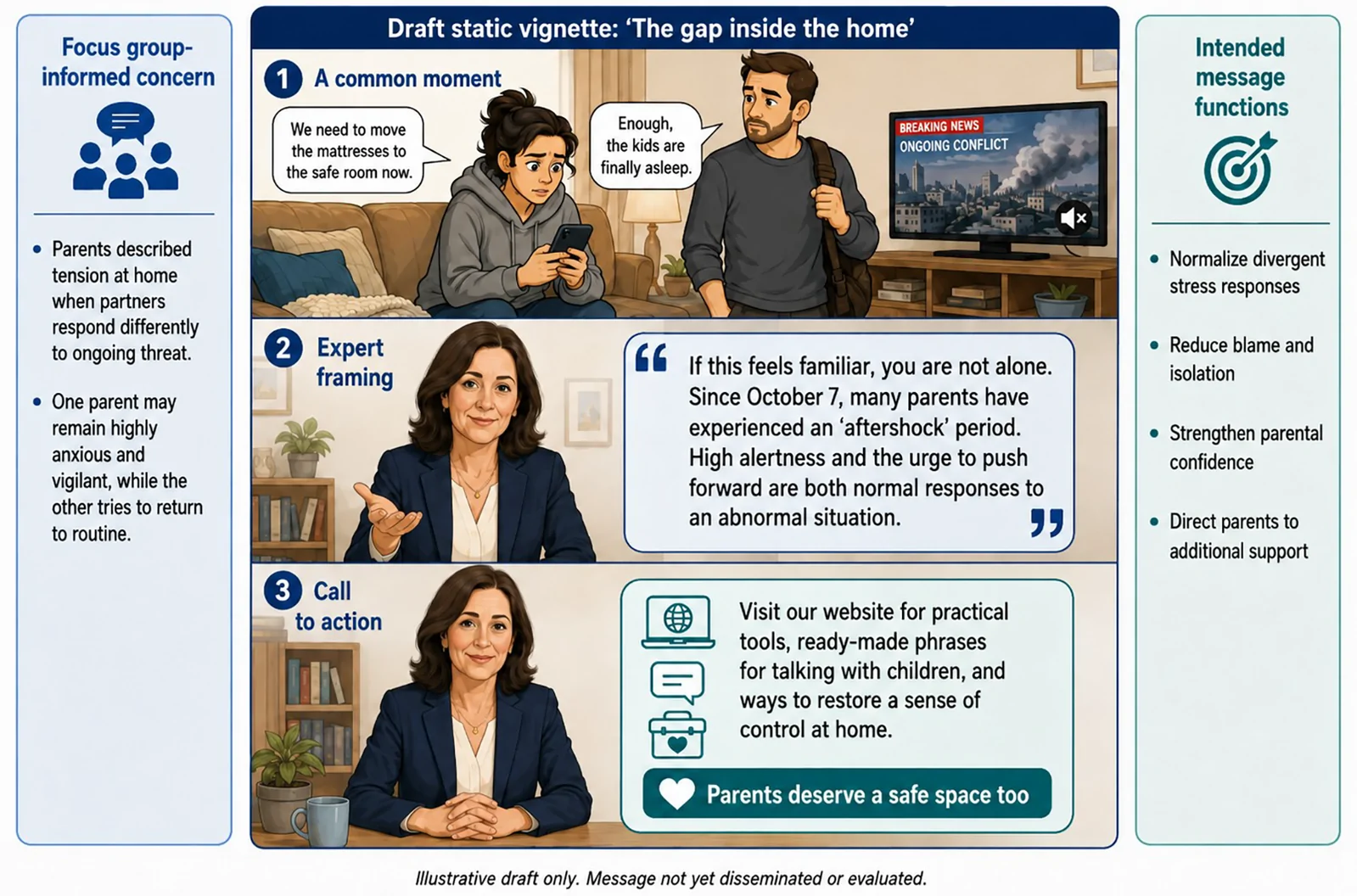
Illustrative draft vignette developed from preliminary focus group-informed message themes. Vignette represents formative message development.

Rather than relying primarily on long-form psychoeducational materials that require active information-seeking, the universal messaging strategy prioritizes short, “bite-sized” content that can be passively encountered through trusted channels embedded in parents’ daily routines. As noted in **Figure 1**, these messages will be available in the multiple languages spoken by different ethnic and racial groups in Israel (i.e. Hebrew, Arabic, Russian and Amharic). Planned channels for dissemination include community WhatsApp groups, Facebook, Instagram, local radio, local and digital billboards, school communications, social media, and other community-based platforms identified through the formative work. The planned dissemination strategy reflects the assumption that parents living under CTS may have limited emotional and cognitive bandwidth, and therefore require support that is easy to encounter, easy to understand, and easy to act on.

Because the universal messaging component is currently under development, the present paper describes the process as a case example rather than as an efficacy trial. No messages have yet been disseminated or evaluated. Once message development and review have been completed, the messages will be disseminated through the planned channels and examined in a follow-up study. The planned evaluation will assess outcomes including visits to the project website, trust in the messaging platform, and participants’ emotional responses following exposure to messages. Accordingly, the materials described here should be understood as formative products of an ongoing universal message-development process, rather than as implementation or effectiveness findings.

### Self-directed online program

Our focus group findings indicated that parents exposed to CTS are seeking a variety of parenting resources to support children during this highly stressful period. For this reason, we developed ADAPT-Israel online, a self-directed parenting program adapted from the original ADAPT online (18). The original ADAPT online program was developed for US military families (18) but has since been extended for other families exposed to traumatic stressors including refugee and migrant families (53, 58), and families with a parent who is exposed to potentially traumatic events in their daily work as a first responder (firefighter, police officer or paramedic). ADAPT-Israel online was modified in collaboration with local experts, and completely new materials were developed. Specifically, while the original ADAPT online program includes 12 modules, each incorporating 1–2 videos each 5–10 minutes in length demonstrating key parenting skills, along with handouts and mindfulness exercises, the ADAPT-Israel online program is far more interactive. Each module includes two short video clips (of 2–3 minutes) interspersed with interactive exercises in which parents apply what they have learned from the videos to daily parenting situations. The program also provides a digital diary in which parents are encouraged, via reflection prompts, to report on their skill practice at home. Parents may access ADAPT online anytime, anywhere; they may do so independently or choose to engage AI live avatar video support to navigate the program. In the latter option, parents enter a virtual family center where they are guided through the ADAPT program with the support of a video avatar who presents and debriefs each video clip and module, offering parents an opportunity to discuss, ask questions about the skills before they try them at home, and troubleshoot any challenges when they return to the ADAPT online avatar-assisted course. The Israel ADAPT online intervention and platform is complete; beta testing of the program is underway, with program rollout planned in late 2026.

### Selective and indicated prevention components of the ADAPT-Israel program

Selective prevention refers to interventions developed and tested for families whose circumstances place their children at greater risk for emotional and behavioral challenges. In the current case of CTS in Israel, approximately 100,000 reservists who are parents of dependent children have been called to active reserve military duty since October 2023; a significant proportion of the military-eligible population (25, 59). The burden of reserve duty is uneven, with highly skilled and/or highly trained individuals (e.g. pilots, infantry soldiers, commanders across all branches of the military, and those in elite units) being called for reserve duty extensively, sometimes for months at a time. While there are many other families in challenging situations in reference to CTS (e.g. those who live in areas hard hit by missile attacks, those with loved ones kidnapped or killed or injured, those evacuated from border areas deemed unsafe for daily life) the focus of our current efforts is reservist families. With the exception of the population of the northern border, reservists represent the largest segment of the population affected by CTS post-October 7^th^ 2023 (and at the time ADAPT-Israel started, many of the families evacuated from the northern border towns had not returned to their homes and thus serving these families was logistically very challenging). We also opted to serve reservist families rather than those living in the communities on the Gaza border or survivors of the terror attacks on October 7^th^ (e.g. those who survived the Nova festival) because more resources were provided for these populations immediately following October 7^th^ (compared with reservist families). We also felt that survivors of the terror attacks might have greater therapeutic needs than the ADAPT parenting group could provide.

In partnership with local authorities, local Non-Governmental Organizations/NGOs, and grassroots community organizations, ADAPT-Israel provides weekly, 14-session face-to-face parenting groups for families in which a parent has been away from the home on reserve military duty for more than 200 days in the post October 7, 2023 conflicts. These parenting groups are similar in structure and approach to the original ADAPT group program in the US, with adapted content for the Israeli CTS context.

#### Early implementation experience: feasibility and acceptability

Three selective-tier 14-week groups were first piloted in 2025 in two cities in Israel (2 groups were held in a town in the center of the country, and one in a town on the coast) and delivered free of charge to participating reserve-parent families in partnership with municipalities and local NGOs. In total 42 parents (24 mothers and 18 fathers) from 26 families participated in the three 14-week groups. All participating parents were married and aged 30–40 years. Participating families had an average of 3.12 children (SD = 1.07), and their children’s mean age was 6.58 years (SD = 2.54). At least one parent in each family had completed more than 200 days of reserve military service since October 7, 2023, with reserve-service duration in the sample ranging from 200 to more than 500 days. Families were recruited through convenience and purposive sampling via municipal and local-authority contacts, community organizations, and targeted WhatsApp groups for reserve families.

Groups were scheduled in the evenings (8-10pm) in local community centers; childcare was not provided, but coffee and snacks were offered. Despite being held during ongoing military conflict (with group meetings disrupted several times due to missile sirens), 81.3% of families completed the program. Exploratory analyses indicated that mothers attended slightly but not significantly more sessions than fathers (M = 70.1% vs. 68.6%; t(40) = 0.19, p = .850, d = 0.06). Attendance proportions also did not differ significantly by family size (1 or 2 vs. 3 or more children; mothers, t(22) = −0.07, p = .948, d = −0.03; fathers, t(16) = −0.19, p = .855, d = −0.09) or by average child age (≤ 6 vs. > 6 years; mothers, t(19.06) = −0.94, p = .361, d = −0.37; fathers, t(16) = −1.00, p = .330, d = −0.47). Given the small pilot sample, these analyses were underpowered to detect any but large effects and should be interpreted with caution.

Parents were asked to complete anonymously the 20-item ADAPT Parent Evaluation Form (see Appendix A for questions) at selected sessions up to five times throughout the intervention, Sessions 1, 4 or 5 or 6, 8 or 9 or 10, 12 and 14. We used the original ADAPT validated satisfaction surveys (42), translated and back translated into Hebrew. The survey items load onto three factors: positive group experience (8 items; e.g., “the group leader encouraged participation” and “I felt accepted by the group”). Participant Satisfaction/Session Content (3 items; e.g., “agreed with the ideas presented”, “information was helpful”) and home practice satisfaction (4 items; e.g., “home practice was helpful”, “assignment fit well with family life”). Positive Group Experience ratings were consistently high across occasions, with means ranging from 4.38 to 4.61 on the 1–5 scale. Participant Satisfaction ratings ranged from 3.93 to 4.70, whereas Home Practice Satisfaction was more moderate and variable, ranging from 3.31 to 3.91. Because questionnaires were anonymous and responses could not be linked across occasions, these values represent occasion-specific evaluations rather than within-participant change over time.

To further examine participants’ experiences with the program, three post-intervention focus groups were conducted via Zoom, one for each intervention site, with a total of 11 mothers who had completed the intervention. Each approximately 90-minute focus group followed a semi-structured guide comprising 10 open-ended questions addressing overall program satisfaction, the perceived usefulness of specific ADAPT components, barriers to participation, and recommendations for improving future groups. Audio recordings were transcribed by a graduate student in social work, de-identified, and segmented into thematic utterances, which served as the unit of analysis.

Transcripts were analyzed using question-guided quantitative content analysis (QCA; 60, 61), organized around six areas: program experience, usefulness of program components, implementation and continued use, perceived relational changes, missing content, and contextual fit. Responses were coded by broad program topic, specific practice/component, and response classification. Inter-rater reliability was assessed on a stratified sample of approximately 20% of the 171 substantive coded references (*n* = 35), with 94.3% agreement (κ = .94) for specific practice/component and 91.4% (κ = .90) for response classification; discrepancies were resolved through discussion.

The focus groups yielded 202 coded references, including 171 substantive references. Mothers particularly valued peer connection, normalization, mutual learning, and facilitator support, and described encouragement, problem solving, emotion-focused content, active listening, and mindfulness/noticing practices as useful. Some practices, including incentive charts, Time-out, and structured home practice, were described as more difficult to implement or sustain. Mothers also reported perceived relational changes and identified areas for further contextual refinement, particularly repeated reserve-service cycles, transitions between solo and shared parenting, and parenting under ongoing threat. While these are small and exploratory findings, the combined data from focus groups and survey ratings suggest that the program was generally viewed positively by participants while also pointing to areas that may benefit from further contextual refinement.

### Indicated prevention: individual telehealth support

The most intensive tier of ADAPT-Israel is delivered individually, via telehealth, with one facilitator working with one family for 14 weekly hourlong sessions. This one-to-one format, which was evaluated as one of the delivery formats in the US comparative effectiveness trial (43), is intended for families who are struggling most, whether with their own parenting or with significant behavioral and emotional difficulties in their children, and who need more individualized attention than a group can provide. Delivering this tier remotely removes geographic and scheduling barriers that are especially acute under conditions of active war, when travel, childcare responsibilities, and reserve service constrain families’ availability.

Thus far, we have piloted the individual telehealth support with three families, comprising six individuals each family with 2–4 children. The parents’ mean age was 37.17 years (SD = 4.88), and the children ranged in age from 2 to 12 years, with a mean age of 6.11 years (SD = 3.06). In all three families, the fathers were officers who had completed 400 days or more of reserve service, including extended and unpredictable absences from home. The families experienced additional stressors associated with the war and reserve service, including employment disruption, the loss of close friends in combat, and continuing uncertainty related to future call-ups. Parents informally reported children’s separation difficulties, emotional dysregulation, angry outbursts, emotional eating, learning difficulties, and regression in developmental tasks such as toilet training. Parents also reported heightened fears for safety among all family members. At the parenting level, families described increased stress, reduced patience, disrupted routines, difficulties maintaining consistent boundaries, and challenges in presenting a united parenting front. The individual format allowed the content and practice of ADAPT parenting skills to be tailored to each family’s circumstances, parenting goals, and children’s specific developmental and emotional needs.

During the final sessions, parents were invited by their facilitator to provide oral feedback about their experiences with the program. In all three families, parents described gaining practical parenting tools and highlighted the value of dedicated time to focus on their parenting, and consider how to respond more effectively during challenging situations. Some parents described responding more calmly and intentionally, while two families noted benefits for their ability to work as a united parenting front. Parents also characterized the telehealth sessions as supportive, nonjudgmental, flexible, and responsive to their families’ circumstances. Several expressed an intention to continue using selected tools and program materials after the intervention ended. These comments represent informal qualitative feedback provided to the facilitator rather than findings derived from a standardized or systematically administered measure.

## Discussion, practical implications, lessons learned and next steps

The ADAPT-Israel case study illustrates how a population-level, parenting-centered public health model can be developed in a society living under continuous traumatic stress. Where the dominant response to widespread trauma is often to expand individual clinical treatment, ADAPT-Israel begins from a different premise: that when threat is ongoing and affects whole communities, an efficient and low stigma way to protect children’s mental health is to support parenting. The approach starts from a high-reach universal layer to more intensive selective and indicated services, matching the intensity and format of support to what families need and want.

While the universal messages are still under development, feedback from parents via focus groups, as well as recent studies from other research groups indicate that parents want and need light-touch support and reassurance from trusted sources during CTS (62–64). As regards the ADAPT parenting groups, preliminary feasibility and acceptability data are encouraging. Families managing extended reserve service, work, and childrearing during active war nonetheless attended groups regularly and were retained throughout the program, attesting to their desire for parenting support. Indeed, the post-intervention focus group data, though conducted with a small sample of attendees, attests to the value parents attributed to the group and the skills taught.

Several practical lessons have emerged. First, community engagement and culturally grounded framing were preconditions for feasibility: beginning with formative focus groups ensured that the universal messages were grounded in parents’ own expressed needs, and partnering with municipalities and local NGOs gave the groups credibility, referral pathways, and physical space. Second, workforce scarcity is a defining constraint under CTS, when demand surges as providers are themselves affected; a train-the-trainer architecture and the use of technology-assisted, self-directed and telehealth formats both build local capacity and extend reach beyond what a clinician-delivered model alone could achieve. Third, delivering support in multiple formats and languages is likely essential for population reach in a diverse society, and multi-format delivery also respects that families under acute stress have limited and fluctuating bandwidth. Fourth, embedding the program in existing municipal infrastructure, with local cost-sharing, offers a more feasible route to sustainability than standalone, grant-dependent services.

Conceptually, ADAPT-Israel reframes the goal of intervention under conditions of unresolved threat. Rather than aiming primarily to provide support for past experiences of traumatic stress, the objective is to sustain adaptive family functioning within an ongoing stress context, using parenting as a single, accessible entry point to benefit both children and parents. This reframing, and the tiered model that operationalizes it, may be relevant to other settings marked by protracted conflict, forced displacement, or chronic community violence, in which mental health systems face the same mismatch between overwhelming need and limited specialist capacity. The core proposition, that population mental health under CTS may be better served via a family-based prevention public health approach than by scaling individual treatment alone, is testable, and we offer ADAPT-Israel as a first case from which such a model can be evaluated and refined.

## Conceptual and methodological limitations

First, the evaluation evidence reported here is preliminary: feasibility and acceptability data come from small pilot groups and rely on participant self-report of satisfaction and not clinical or behavioral outcomes. Without a comparison condition, or randomized design we cannot support or effectiveness claims. The program’s evidence base derives from US military family trials, and the assumption that these benefits will transport to the Israeli CTS context, though theoretically grounded, remains to be demonstrated. Second, both the universal messaging component and ADAPT-Israel’s online program are still under development (currently in beta testing).

Third, the selective and indicated tiers have so far reached a tiny proportion of one segment of the affected population, families of long-serving reservists, and have not yet served other highly exposed groups, including evacuated, bereaved, and directly attacked families, whose needs may differ. Finally, the multilingual and multi-format reach to which the model aspires, including Arabic, Amharic, and Russian versions of the online program, is still being built out. We report this case at an early stage precisely so that these limitations can guide the more rigorous evaluation now being planned.

## Summary and next steps

Building on this experience, there are several next steps. The program is being scaled through two mechanisms designed for sustainability under conditions of workforce scarcity: a train-the-trainer model, in which experienced facilitators become trainers and coaches, and a municipal cost-sharing partnership model, with staged expansion of facilitator training and groups across additional local authorities. Six facilitators were trained between January and March 2026 and are currently delivering an additional three groups with a total of 24 families across the groups. We are currently in discussions with both NGOs and municipal and governmental agencies to plan for a full-scale implementation of the ADAPT intervention across Israel, together with expansion of eligibility from reserve families to the broader population of families exposed to CTS.

Second, we are planning an evaluation of ADAPT Israel via two separate studies. The first is a three group non-inferiority randomized controlled trial/RCT to compare ADAPT-Israel online and ADAPT groups with a message-only control group. The second will test the messaging campaign through rigorous evaluation of message dissemination to discrete populations in different areas of the country.

Taken together, ADAPT-Israel represents, to our knowledge, the first attempt to reframe support for families under continuous traumatic stress as a tiered, parenting-centered public health system that meets families at the level of intensity they need and want. What we have described, a beginning: a program built within the communities it serves, a universal messaging layer and online program still taking shape, and early feasibility and acceptability data drawn from small, self-selected pilot groups. Early signals are nonetheless encouraging - families juggling reserve service, work, and childrearing under active bombardment came, remained, and told us the groups benefited them. These data lend weight to a proposition that now warrants rigorous evaluation: that when threat is chronic and community-wide, protecting children may depend less on individual treatment than on strengthening the caregiving relationships that surround them.

This work is urgent because Israel is not the only country with a population living under conditions of CTS. From Ukraine to other settings of protracted conflict and displacement, families are raising children amid ongoing threat, and mental health systems everywhere confront the same mismatch between overwhelming need and limited specialist capacity. We offer ADAPT-Israel as a first case for a model we hope that other communities living under chronic threat might one day make their own.

## Data Availability

The raw data supporting the conclusions of this article will be made available by the authors, without undue reservation.
